# Neutrophil Extracellular Traps in Pancreatic Ductal Adenocarcinoma: A Vicious Cycle in the Tumor Microenvironment and Targeted Interventions

**DOI:** 10.7150/ijbs.133035

**Published:** 2026-05-11

**Authors:** Lin Zhao, Haodong Yuan, Zixiang Chen, Xueli Xia, Xinyu Tian, Shengjun Wang

**Affiliations:** 1 Department of Laboratory Medicine, Jiangsu Province Engineering Research Center for Precise Diagnosis and Treatment of Inflammatory Diseases, Affiliated Hospital of Jiangsu University, Zhenjiang 212002, China.; 2 Department of Immunology, Jiangsu University School of Medicine, Zhenjiang 212013, China.; 3 Department of Laboratory Medicine, Nanjing Drum Tower Hospital, Nanjing University Medical School, Nanjing 210008, China.

**Keywords:** neutrophil extracellular traps (NETs), pancreatic ductal adenocarcinoma (PDAC), tumor microenvironment (TME), interventions

## Abstract

Neutrophil extracellular traps (NETs) are web-like structures released by activated neutrophils and initially identified for their role in antimicrobial defense. In recent years, growing evidence has demonstrated that NETs contribute to the development and progression of various malignancies. Pancreatic ductal adenocarcinoma (PDAC) is a highly aggressive digestive-system cancer characterized by strong invasiveness and poor prognosis. Notably, substantial infiltration of NETs is frequently observed within the PDAC tumor microenvironment (TME). Clinical evidence indicates that this phenomenon is closely associated with metastatic progression and reduced patient survival. This review systematically elaborates how the PDAC TME recruits and activates neutrophils and induces NET formation through multiple pathways, including extracellular matrix (ECM) signaling, cell-cell interactions, cytokine secretion, and epigenetic dysregulation. In addition, it examines the mechanisms through which NETs, functioning as a regulatory hub, facilitate PDAC progression by inducing angiogenesis, altering the stromal structure, driving tumor cell proliferation and invasion, and creating an immunosuppressive environment. Finally, we summarize the latest therapeutic strategies targeting NETs in PDAC and provide an outlook on future research directions in this field.

## 1. Introduction

Pancreatic ductal adenocarcinoma (PDAC) is the most common histological subtype of pancreatic cancer, accounting for more than 90% of all pancreatic tumors and represents one of the most aggressive and lethal malignancies in the gastrointestinal tract 1, 2. Statistical data indicate that the overall 5-year survival rate for PDAC patients is approximately 13% 3. Globally, while the incidence of PDAC ranks around 12th among all cancers, its cumulative mortality rate is as high as 6th 4. Although significant variations in PDAC incidence exist across countries, the overall trend shows a steady annual increase in global prevalence 5-7. In recent years, despite certain advancements in therapeutic strategies and treatment efficacy for PDAC, the overall prognosis for patients remains unsatisfactory.

In PDAC, neutrophil infiltration persists at elevated levels and is closely associated with poor patient prognosis 8. Clinical studies have further established that the peripheral blood neutrophil-to-lymphocyte ratio (NLR) is a significant independent predictor of PDAC prognosis 9-11. Notably, neutrophils not only mediate immune responses through classic mechanisms such as phagocytosis and degranulation, but also play a crucial role in various physiological and pathological processes by forming neutrophil extracellular traps (NETs). Although NETs were initially discovered in sepsis models and extensively studied 12, recent research has shown that NETs are also involved in the development, progression, and metastasis of various malignant tumors 13-17. In 2019, a study reported that tumor-infiltrating NETs could serve as an independent predictor of postoperative prognosis for PDAC patients. Their research further demonstrated that incorporating NETs into the tumor-node-metastasis (TNM) staging system significantly improves the accuracy of prognostic stratification for PDAC 18. Additionally, the development of single-cell RNA sequencing (scRNA-seq), spatial transcriptomics (ST), and multi-omics methods has provided deeper mechanistic insights into NET-mediated tumor-stroma interactions and supports their potential as biomarkers for disease monitoring and prognosis assessment 19-21. Recent reviews have enhanced our understanding of the role of NETs in pancreatic diseases 22. However, given that PDAC exhibits significant features of fibrosis, immunosuppression, and treatment resistance, treating NETs as downstream inflammatory by-products is insufficient to fully explain their functional contribution to disease progression.

In this review, we provide a systematic elucidation of the upstream regulatory signals within the PDAC tumor microenvironment (TME) that trigger NETs formation, along with the downstream effector functions mediated by NETs, and aim to establish an integrated framework to delineate the intricate interplay between the TME, NETs, and tumor progression in PDAC. Specifically, we focus on how PDAC-related microenvironmental factors drive NET formation via specific molecular pathways, and how NETs, in turn, promote tumor progression through mechanisms such as matrix remodeling, immunosuppression, angiogenesis, metastasis, thrombosis, and treatment resistance. By incorporating these findings into a PDAC-specific theoretical framework, this review aims to elucidate the mechanisms and translational implications of NETs in PDAC.

## 2. Biology of NETs: Composition, Function, and Formation Mechanisms

The formation of NETs, termed NETosis, represents a distinct form of cell death that differs from apoptosis and necrosis 23. It is characterized by the release of decondensed chromatin and granular contents into the extracellular space, reflecting a unique death program of neutrophils 24, 25. However, extracellular trap (ET) formation is not restricted to neutrophils. Accumulating evidence suggests that other granulocytes, including eosinophils and basophils, can also form ETs (EETs and BETs) and mediate similar biological functions 26, 27. These observations indicate that ET formation may represent a broader and evolutionarily conserved biological phenomenon. A comparative overview of NETs, EETs, and BETs is provided in **Table 1**. Among these, NETs remain the most extensively studied.

### 2.1 Composition and Functional Implications of NETs

#### 2.1.1 Composition of NETs

In 2004, Brinkmann V and colleagues published a seminal paper in the journal Science. This paper provided the first systematic elucidation of the structure and function of NETs. Their research demonstrated that NETs are complex extracellular web-like structures composed of DNA, histones, and various granular proteins 12. Initially, NETs were recognized as a unique form of cell death capable of capturing and eliminating pathogens. Subsequent studies have demonstrated that the composition of NETs varies in a stimulus-dependent manner. Proteomic analyses showed that NETs induced by different stimuli exhibit significant heterogeneity. These stimuli include lipopolysaccharide (LPS), phorbol 12-myristate 13-acetate (PMA), and calcium ionophore A23187. Both protein composition and post-translational modification profiles vary accordingly. These findings suggest that NETs formed under different conditions may perform distinct biological functions 28, 29. Currently, commonly used markers for identifying NETs include the DNA component of NETs (NET-DNA), citrullinated histone H3 (CitH3), and myeloperoxidase (MPO) 30-32.

#### 2.1.2 Functional Implications of NET Components

The biological functions of NETs are closely related to their molecular composition, and this composition-dependent functional diversity is particularly evident in the TME. NET-DNA plays an important regulatory role in tumor progression. For instance, NET-DNA can bind to the coiled-coil domain containing 25 (CCDC25) receptor on the tumor cell membrane, activate the β-parvin-RAC1-CDC42 signaling pathway, induce cytoskeletal rearrangement, and promote the directed migration of tumor cells 14. Additionally, in hepatocellular carcinoma (HCC), studies have shown that NET-DNA can bind to the transmembrane and coiled-coil domains 6 (TMCO6) receptor on CD8⁺ T cells, thereby inhibiting the anti-tumor immune response and promoting disease progression 16. Further research reported that neutralizing the negative charge of NET-DNA with positively charged polyamino acids can block its interaction with CCDC25, thereby inhibiting tumor metastasis 33.

In addition to the NET-DNA component, its protein component also plays a crucial role in tumor regulation. As a key effector molecule released during NET formation, neutrophil elastase (NE) promotes the migration of colorectal cancer (CRC) cells through the extracellular signal-regulated kinase (ERK) signaling pathway. Further, in a CRC liver metastasis mouse model, inhibiting NE activity significantly reduced the formation of CRC liver metastases, highlighting its important role in tumor metastasis and its potential as a therapeutic target 34. Both *in vitro* and *in vivo* experiments in mice have shown that the serine protease associated with NETs, cathepsin G (CTSG), can promote tumor invasion and metastasis and is closely associated with the poor prognosis of HCC 35. Meanwhile, MPO contributes to oxidative stress by regulating reactive oxygen species (ROS) levels and may play a role in tumor progression and therapy resistance 36. CitH3 is a key structural and characteristic marker of NETs, widely used for NET detection and associated with disease progression. Notably, in extrahepatic cholangiocarcinomas (EHCCs), CitH3-positive NETs are significantly associated with poor postoperative survival and can independently predict patient outcomes, highlighting the potential role of NET-related components in tumor progression 37, 38. Additionally, programmed death-ligand 1 (PD-L1) has been reported to be associated with NET structures, where it may interact with programmed cell death protein 1 (PD-1) on tumor-infiltrating lymphocytes (such as CD4⁺ and CD8⁺ T cells), thereby contributing to T cell exhaustion and dysfunction, although its precise localization within NETs remains unclear 39. NET-derived high mobility group box 1 (HMGB1) can promote tumor metastasis by inducing epithelial-mesenchymal transition (EMT) 40, while arginase-1 (ARG1) enhances immunosuppression through its interaction with cathepsin S (CTSS), thereby facilitating PDAC progression 41.

More and more evidence has gradually clarified the crucial role of NETs and their constituent molecules in disease progression, especially in tumor progression **(Table 2)**. Although significant progress has been made in this field, the heterogeneity of NETs themselves and their complex composition still, to some extent, limit the systematic understanding of the mechanism by which they drive tumor progression. Especially in the context of PDAC, a highly heterogeneous tumor, the further development of targeted therapy research may rely on a more in-depth and systematic analysis of the mechanism of action of NETs and their key components during the disease process.

### 2.2 Mechanisms of NET Formation

**Figure 1:**
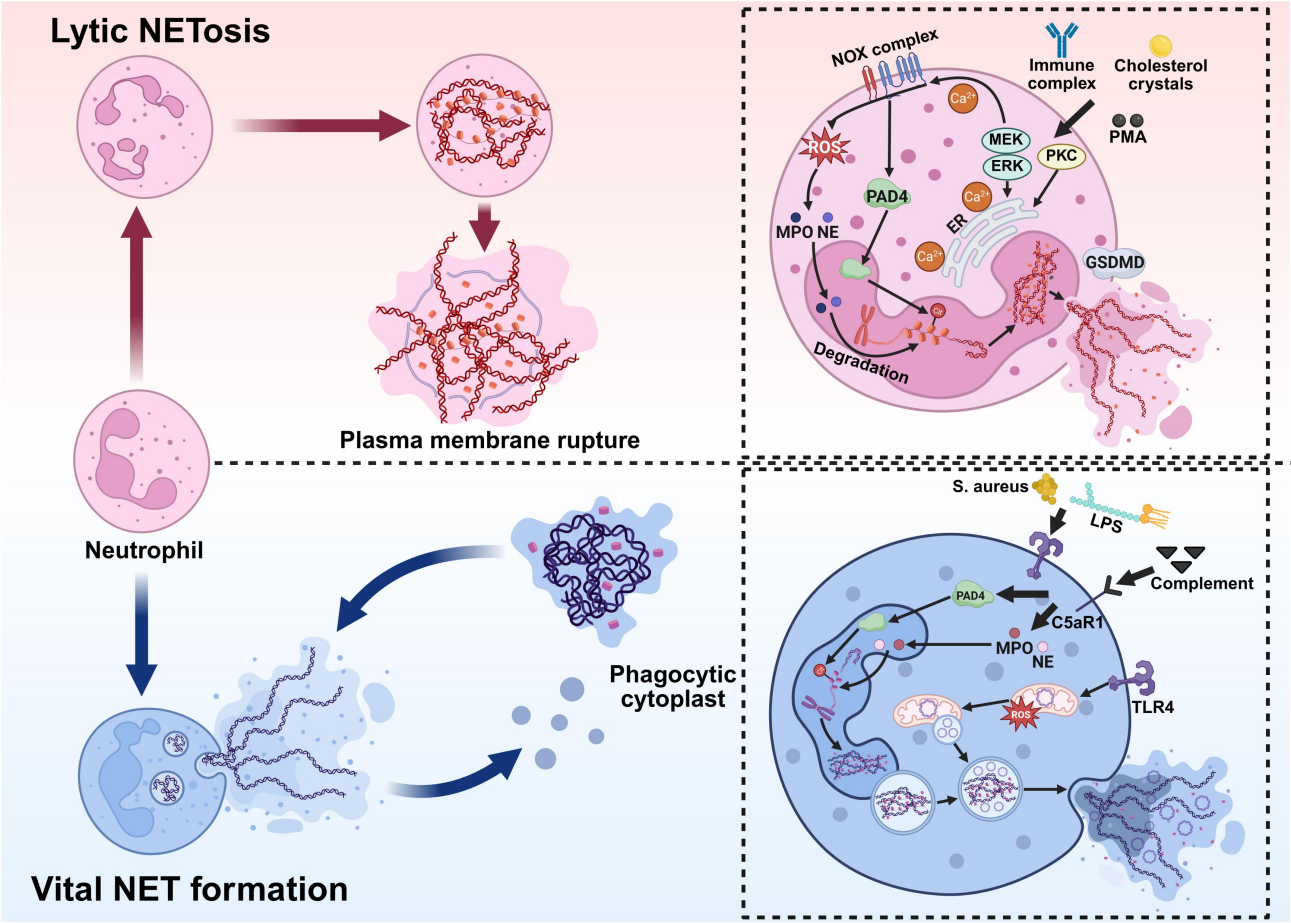
** Pathways of NET formation. NETs are formed through two distinct mechanisms: lytic NETosis, which results in neutrophil death, and vital NET formation, which preserves cell viability. Lytic NETosis.** Stimuli such as immune complexes, cholesterol crystals, or PMA activate the NOX complex, triggering robust ROS production. This is accompanied by dynamic Ca²⁺ fluxes and activation of PKC and the Raf-MEK-ERK pathway, which promote PAD4-mediated histone citrullination and chromatin decondensation. MPO and NE then translocate to the nucleus to degrade chromatin and associate with granular proteins. Finally, GSDMD-dependent plasma membrane rupture releases these components into the extracellular space, forming NETs, ultimately resulting in lytic cell death. **Vital NET Formation.** Pathogens (e.g., *Staphylococcus aureus*), LPS, or complement signals activate neutrophils through pathways involving TLR4 and C5aR1. ROS (primarily of mitochondrial origin) and PAD4 participate in chromatin decondensation. The chromatin is then transported via vesicular structures and released extracellularly through exocytosis as NETs composed of DNA, MPO, and NE. This process preserves plasma membrane integrity and maintains neutrophil viability. *Created with BioRender.com.

Regarding the mechanisms of NET formation, two primary models have been proposed: lytic NETosis and vital NET formation** (Figure 1)**.

#### 2.2.1 Lytic NETosis Formation

During the lytic NETosis pathway, neutrophils undergo a series of highly programmed biological events in response to stimulation. This process is typically initiated within 1-4 hours after activation and is characterized by nuclear envelope disassembly, chromatin decondensation, the integration of decondensed chromatin with cytoplasmic proteins, and the eventual release of NETs following plasma membrane permeabilization 42, 43. Therefore, the lytic NETosis pathway is commonly referred to as “suicide NETosis” NETs can be triggered by various stimuli, including PMA, bacteria, antibodies, *Cryptosporidium*, cholesterol crystals, and interleukins 29, 44-48. Notably, host-derived cytokines also contribute to NET formation; for example, histamine can induce NETs in a dose-dependent manner via the NADPH oxidase (NOX)/ERK/p38 pathway 49. These stimuli activate multiple signal transduction pathways, leading to increased intracellular calcium concentration and activation of NOX. Subsequently, NOX catalyzes the generation of ROS from molecular oxygen, thereby initiating downstream events. ROS serve as central mediators of chromatin decondensation in neutrophils. Their accumulation and mode of action directly determine the release of NETs 50-52. In resting neutrophils, NE is primarily stored in azurophilic granules, which are involved in phagocytosis. Part of NE is bound to MPO and attached to the granule membrane, while the remainder resides within the granule lumen. ROS production activates NE and promotes its release from the MPO-containing azurophilic granule complexes into the cytoplasm. The released NE binds to F-actin and mediates the degradation of actin filaments, thereby disrupting the cytoskeleton. NE then translocates to the nucleus, where it promotes initial chromatin decondensation by partially cleaving histones. Hydrogen peroxide selectively promotes the release of NE into the cytoplasm, a process that requires cooperation with MPO 53-55. Additionally, peptidylarginine deiminase 4 (PAD4), upon calcium-dependent activation, catalyzes histone citrullination, weakening histone-DNA interactions and further driving extensive chromatin decondensation. Following nuclear envelope disintegration, decondensed chromatin is released into the cytoplasm 42, 56. During the terminal stage of NET release, increased plasma membrane permeability is a key step in successful NET extrusion. Recent studies show that the pore-forming protein gasdermin D (GSDMD) plays a central role in the formation of lytic NETs. By forming pores in the plasma membrane, GSDMD increases membrane permeability, which facilitates the extrusion of chromatin-granule complexes and the final release of NETs 57.

#### 2.2.2 Vital NET Formation

Vital NET formation refers to a rapid, NOX-independent mechanism by which neutrophils release extracellular DNA webs without undergoing lytic cell death. In contrast to classical (or lytic) NETosis, which culminates in plasma membrane rupture and cell death, neutrophils remain viable following vital NET release and retain key immune functions, including chemotaxis, phagocytosis, and cytokine secretion 58, 59.

This process is typically induced by physiological stimuli common to early immune responses, such as bacterial components, platelet-neutrophil interactions, and inflammatory mediators, including LPS, complement component 5a (C5a), and granulocyte-macrophage colony-stimulating factor (GM-CSF). C5a primarily signals through complement C5a receptor 1 (C5aR1) on neutrophils, thereby promoting neutrophil activation and NET formation. Notably, within the vasculature, platelets can rapidly induce NET release via Toll-like receptor 4 (TLR4)-mediated signaling, underscoring the importance of vital NETs in intravascular immune defense 60. A key feature of vital NET formation is the diversity of DNA sources. While classical NETosis primarily involves nuclear DNA, vital NETs can consist of either nuclear or mitochondrial DNA (mtDNA), depending on the stimulus. Studies have shown that upon stimulation with cytokines or immune complexes, neutrophils can selectively release mtDNA to form NETs with minimal alteration in nuclear morphology 61. Mechanistically, vital NET formation is characterized by limited chromatin decondensation and vesicular-mediated DNA transport. Upon stimulation, nuclear or mitochondrial DNA complexes with histones and antimicrobial proteins from neutrophil granules. Then, the DNA-protein complex is packaged into cytoplasmic vesicles. These vesicles traffic to and fuse with the plasma membrane, releasing NET structures into the extracellular space via exocytosis without compromising membrane integrity 58. Unlike lytic NETosis, this process does not require massive ROS generation by NOX, extensive nuclear envelope disintegration, or full chromatin expansion 62, 63.

Chronic granulomatous disease (CGD) provides an important human disease model supporting the concept of vital NET formation. CGD is a primary immunodeficiency most commonly caused by mutations in genes encoding subunits of the NOX complex, which is essential for phagocytic ROS generation 64. Although CGD neutrophils lack functional NOX and cannot mount a conventional respiratory burst, they retain the ability to form NETs 65. Interestingly, despite the absence of functional NOX, NET formation has been observed in patients with CGD. For instance, peroxisome proliferator-activated receptor-gamma (PPAR-γ) agonists such as pioglitazone and rosiglitazone induce mitochondrial ROS production and subsequently trigger NET formation in a NOX-independent manner in CGD neutrophils 66, 67.

### 2.3 Functional Implications of NET Heterogeneity in PDAC

NET heterogeneity reflects differences in formation pathways, molecular composition, and functional properties. In tumors, distinguishing between lytic NETosis and vital NET formation is particularly informative, as these processes are likely to generate distinct downstream effects and functional states within the TME. Lytic NETosis is typically associated with extensive chromatin release, neutrophil death, and a pronounced inflammatory response, together with the release of proteases and damage-associated molecular patterns (DAMPs) 25, 68. This form of NET release has been linked to tissue injury, ECM remodeling, and the development of tumor-supportive niches. The accumulation of NET-associated components may also contribute to immunosuppression and facilitate tumor cell dissemination. By contrast, vital NET formation occurs more rapidly and in a regulated manner, allowing neutrophils to remain viable and retain key cellular functions 60. In some settings, this process may support immune surveillance or temporarily restrain tumor growth 14. However, in the context of persistent inflammation or tumor-derived signals, vital NETs may likewise contribute to the establishment of an immunosuppressive TME.

In PDAC, it remains unclear whether specific stimuli preferentially drive lytic NETosis or vital NET formation, or whether these distinct NET states have distinct effects on tumor progression. Although NET formation has been widely described in PDAC, studies that clearly distinguish NET subtypes or directly compare their functional roles remain scarce. Notably, much of what is currently known about NET heterogeneity comes from non-PDAC models, and its relevance in PDAC remains to be validated. Future work should therefore focus on defining how different NET formation pathways contribute to PDAC progression, which may help refine current models and inform more targeted therapeutic strategies.

## 3. Mechanisms of NET Formation in the PDAC TME

The TME is a complex and dynamic ecosystem surrounding tumor cells. It is primarily composed of cancer cells, adjacent immune cells, blood vessels, the ECM, fibroblasts, lymphocytes, bone marrow-derived inflammatory cells, and various signaling molecules 69-72. The formation of the TME relies on dynamic interactions between cancer cells and host components, and it plays a critical role in tumor initiation, progression, therapeutic resistance, and metastasis 73.

### 3.1 Characteristics of the TME in PDAC

Among all solid tumors, PDAC has attracted significant attention due to its high malignancy and extremely poor clinical prognosis 74. The TME of PDAC exhibits unique and complex characteristics, with the most prominent being a highly fibrotic stromal response 75, 76. Activated pancreatic stellate cells (PSCs) differentiate into cancer-associated fibroblasts (CAFs), forming a dense stroma. CAFs possess remarkable proliferative and secretory capabilities, enabling them to deposit large amounts of ECM components, such as type I and III collagen, fibronectin, laminin, and hyaluronic acid (HA), forming a dense fibrotic network that accounts for over 80% of the tumor tissue volume 68. This aberrant stroma not only significantly increases the mechanical stiffness and interstitial fluid pressure of the tumor tissue but also severely impedes angiogenesis, restricts drug penetration, and inhibits immune cell infiltration, thereby substantially limiting the efficacy of chemotherapy and immunotherapy 77, 78. Furthermore, CAFs establish a complex bidirectional signaling network with tumor cells by secreting various growth factors, including transforming growth factor-beta (TGF-β), platelet-derived growth factor (PDGF), fibroblast growth factor (FGF), and hepatocyte growth factor (HGF), as well as cytokines such as interleukin-6 (IL-6), thereby collectively promoting PDAC progression and the formation of an immunosuppressive microenvironment 79-83. Notably, single-cell transcriptomic studies have revealed significant functional heterogeneity among CAFs. Certain subpopulations (e.g., PLXDC1^+^ tumor-associated PSCs, TPSCs) exhibit pro-tumorigenic properties, while others may possess the potential to suppress tumor progression. This “dual role” explains the inconsistent clinical outcomes of previous CAF-targeted therapeutic strategies 84. Further research has shown that the activation of PSCs/CAFs involves signaling pathways such as Wnt/β-catenin and EGFR/ERBB2, as well as epigenetic regulatory elements, such as super-enhancers. These findings provide potential new targets for modifying the TME and overcoming treatment resistance 16, 85, 86.

In addition to the fibrotic stroma, strong immunosuppression is another notable feature of the PDAC TME 87. Although PDAC exhibits substantial immune cell infiltration, it is predominantly composed of immunosuppressive cell populations. Tumor-associated neutrophils (TANs), a key immune cell population in the PDAC TME, have recently been recognized as playing a central role in its pathogenesis and immune regulation 88. Similar to how macrophages can polarize into pro-inflammatory, anti-tumor M1 phenotypes or immunosuppressive, pro-tumor M2 phenotypes under specific stimuli, TANs in the TME also exhibit remarkable plasticity. Studies have shown that TANs can also be categorized into anti-tumor N1 or pro-tumor N2 phenotypes 89. Single-cell RNA sequencing (scRNA-seq) studies have revealed that TANs in PDAC exhibit significant functional heterogeneity and plasticity, differentiating into distinct subpopulations in response to microenvironmental signals (e.g., hypoxia, endoplasmic reticulum stress). Among these subsets, terminally differentiated pro-tumorigenic TAN subpopulations are closely associated with poor patient prognosis. These cells are characterized by markedly enhanced glycolytic activity, which is directly regulated by BHLHE40. As a key downstream effector of hypoxia and endoplasmic reticulum stress, BHLHE40 promotes neutrophil polarization toward a pro-tumorigenic TAN phenotype, thereby enhancing their immunosuppressive and tumor-promoting functions. High infiltration of BHLHE40^+^ neutrophils indicates a worse clinical outcome 90. Recent studies have further confirmed that the TME of PDAC exhibits high heterogeneity and complexity, in which an immunosuppressive state coexists with pro-inflammatory signals, collectively promoting tumor progression and therapy resistance 91. Within this context, neutrophils are not only extensively recruited and activated but may also be induced to form NETs through multiple mechanisms 92-94, thereby establishing a mechanistic foundation for their contribution to PDAC progression and therapeutic resistance.

### 3.2 Mechanisms Regulating NET Formation

#### 3.2.1 ECM Signaling

TME is characterized by dense ECM deposition 95. One of the main components of ECM is collagen, and its accumulation in the TME has been widely recognized as a key factor in promoting tumor progression 96-99. Discoidin domain receptor (DDR1 and DDR2), the only receptor tyrosine kinases activated by fibrillar collagen 96, 100, mediate both tumor cell invasion and modulation of the immune microenvironment 101-103. The study by Deng et al. reported that collagen-activated DDR1 triggers the PKCθ/SYK/NF-κB signaling cascade in cancer cells, significantly upregulating the expression and secretion of the C-X-C motif chemokine ligand 5 (CXCL5). High levels of CXCL5 subsequently recruit large numbers of TANs into the tumor region, and these neutrophils, upon activation, form NETs. NETs can capture cancer cells and enhance their migration and invasion capabilities, ultimately promoting distant metastasis in PDAC. Although this study did not dissect downstream signaling pathways in neutrophils, established C-X-C motif chemokine receptor 2 (CXCR2)-dependent signaling pathways that regulate chemotaxis, activation, and ROS production may provide a plausible mechanistic basis for CXCL5-induced NET formation 104, 105. This research reveals the critical role of the DDR1 signaling pathway in linking the collagen-rich microenvironment with immune regulation, offering new insights for targeted therapeutic strategies 102.

#### 3.2.2 Cell-Cell Interactions

Mesothelin (Msln), a glycosylphosphatidylinositol-anchored protein (GPI-AP) normally expressed in mesothelial cells, is markedly upregulated in PDAC cells, constituting the main source of Msln in the TME. Msln binds to the macrophage mannose receptor (CD206) through its GPI-AP mannose residues, modulating macrophage polarization. High Msln expression in patient-derived PDAC samples correlates with increased CD206⁺ macrophages and poor overall survival. In the highly metastatic murine PDAC cell line FC1245, Msln levels were significantly elevated, and tumor-bearing mice inoculated with these cells exhibited substantial enrichment of CD206⁺ macrophages. Further investigation reported that Msln secreted by metastatic cancer cells could induce macrophages to express vascular endothelial growth factor A (VEGFA) and the calcium-binding protein S100A9 93. S100A9 is a potent neutrophil chemoattractant, and high levels of extracellular S100A9 are associated with neutrophil recruitment, activation, degranulation, release of inflammatory cytokines, and formation of NETs 106, 107. Macrophage-derived VEGFA supports cancer cell proliferation and survival through a feedback mechanism, while S100A9 enhances neutrophil infiltration in the lungs and promotes NET formation, thereby further facilitating PDAC metastasis 93. These findings uncover a novel role for Msln in regulating macrophage function and its crosstalk with neutrophils, offering a potential therapeutic target for PDAC.

Additionally, tissue inhibitor of metalloproteinases 1 (TIMP1), a classical regulator of stromal metabolism, is highly expressed in various malignancies, including PDAC, and is closely associated with poor prognosis 108. Mechanistically, TIMP1 binds to the neutrophil surface receptor CD63, activating the ERK signaling cascade to induce NET release. Preclinical studies in PDAC models show that TIMP1 promotes intratumoral NETs, and its inhibition prolongs survival. Clinically, TIMP1 has been reported to co-localize with NETs in tumor tissues, and plasma TIMP1 levels correlate with NET-associated markers, forming a composite signature that may improve patient risk stratification. The combination of TIMP1, NET markers, and carbohydrate antigen 19-9 (CA19-9) has been shown to enhance prognostic stratification in PDAC patients. Mechanistically, the TIMP1/CD63/ERK axis has been implicated in NET-mediated PDAC progression, suggesting a potential therapeutic target. However, current evidence remains limited, and further validation in larger clinical cohorts and functional studies is required 109.

#### 3.2.3 Cytokine Signaling

The liver is the predominant site of PDAC metastasis, contributing to poor prognosis 110, 111. Recent advances in scRNA-seq and ST have provided important insights into the immune composition and functional state of the PDAC liver metastatic microenvironment. By combining scRNA-seq and ST analyses of surgically resected primary and metastatic PDAC samples, Xu W and colleagues reported a distinct neutrophil subpopulation with pro-metastatic features—S100 calcium-binding protein A12-positive neutrophils (neutrophils-S100A12). This subpopulation was specifically localized at the invasive front of metastatic lesions and demonstrated pronounced pro-metastatic functions. Within these invasive regions, inflammatory responses were highly active, and tumor cells exhibited markedly increased aggressiveness 92. The TME was rich in TGF-β, which is known to regulate the pro-tumor functions of neutrophils 112, 113. Further analyses suggested that tumor-derived TGF-β may activate small mother against decapentaplegic (SMAD) family member 3 (SMAD3) signaling in neutrophils, which is associated with increased expression of the transcription factor nuclear factor erythroid 2 (NFE2). NFE2 has been linked to the regulation of PAD4 expression, a key mediator of NET formation. In this context, NET accumulation at the invasive front was associated with enhanced tumor cell invasion and local immunosuppression, suggesting a potential TGF-β/SMAD3/NFE2/PAD4 axis contributing to PDAC liver metastasis 92. However, most evidence supporting this pathway comes from liver metastatic samples and transcriptomic analyses, and it remains unclear whether this mechanism operates similarly in primary PDAC or in other metastatic sites. In addition, although human data provide important translational support, current evidence is stronger for association than for direct causality. Therefore, this axis is best interpreted as one of several context-dependent mechanisms contributing to NET formation in PDAC, rather than a universally established pathway.

Interleukin-17 (IL-17), secreted by CD4⁺ and γδ T cells during pancreatic tumorigenesis, contributes to the initiation and progression of PDAC precursor lesions 114. Studies have shown that upon Kras mutation activation, upregulation of interleukin-17 receptor A (IL-17RA) in epithelial cells enhances stem-like properties in precancerous lesions 114, 115. Further research reported the elevated levels of IL-17 in both spontaneous and orthotopic transplanted PDAC mouse models, as well as detectable expression in human PDAC tissues, whereas it was nearly absent in healthy tissues. Functionally, IL-17 has been associated with immune suppression through promoting neutrophil infiltration and NET formation, while inhibiting the recruitment and activation of CD8⁺ T cells. Blocking IL-17 enhances responses to immune checkpoint inhibitors in a CD8⁺ T cell-dependent manner 94. Notably, most mechanistic evidence linking IL-17 to NET formation derives from mouse models, and its consistency across different experimental systems and in human PDAC remains unclear. In particular, IL-17 may not directly induce NETosis but instead promotes NET formation indirectly by enhancing neutrophil recruitment and reshaping the tumor immune microenvironment. Therefore, IL-17 is more appropriately interpreted as part of a broader inflammatory signaling network that regulates neutrophil behavior and NET formation, rather than a standalone driver.

Clinical studies also suggest a potential link between NETs and PDAC progression. It has been observed that plasma NET levels are significantly elevated in patients following pancreatectomy, which may reflect the systemic inflammatory response triggered by surgical trauma in the early postoperative period. Such inflammation is often associated with increased levels of various pro-inflammatory cytokines, such as interleukin-8 (IL-8), IL-6, and granulocyte colony-stimulating factor (G-CSF), which are believed to be involved in neutrophil activation and NET formation. Furthermore, clinical observations indicate that robot-assisted minimally invasive surgery is associated with lower NET levels, whereas open surgery and postoperative pancreatic fistula are associated with elevated NET levels 116. These findings remain largely correlative, and the extent to which perioperative NET formation directly contributes to disease progression or metastasis remains unclear. Moreover, clinical heterogeneity, including differences in surgical approach and postoperative complications, may confound the interpretation of NET-related biomarkers.

#### 3.2.4 Epigenetic Modification

Recent advances in genome-scale sequencing technologies have underscored the critical role of epigenetic regulation in the development and progression of PDAC. Emerging evidence suggests that epigenetic dysregulation may also contribute to NET formation; however, the precise molecular mechanisms remain to be fully elucidated. Current evidence primarily supports an indirect regulatory model, in which tumor-intrinsic epigenetic alterations remodel the TME, which, in turn, influences neutrophil activation and promotes NET release 117-119. Lysine (K)-specific demethylase 6A (KDM6A), also known as UTX (ubiquitously transcribed tetratricopeptide repeat protein on chromosome X), functions by catalyzing the demethylation of trimethylation of lysine 27 on histone H3 (H3K27me3). It is a key epigenetic factor that maintains chromatin openness and regulates gene expression 120. KDM6A regulates the expression of pluripotency-related and lineage-specific genes, thereby contributing to cell fate determination and the maintenance of cellular identity, and is essential for pancreatic development 121, 122. Notably, KDM6A can escape X-chromosome inactivation in both mice and humans, and its loss has been demonstrated to markedly promote the proliferation, invasion, and metastasis of PDAC. This deficiency is closely associated with a poor prognosis and is a significant independent prognostic factor for the disease 123. Mechanistically, loss of KDM6A has been shown to upregulate the expression of C-X-C motif chemokine ligand 1 (CXCL1), which in turn activates the CXCR2 signaling pathway in TANs. This CXCL1/CXCR2 axis promotes neutrophil recruitment and NET formation, thereby exacerbating tumor-associated inflammation and disease progression. Notably, inhibition of CXCL1 has been reported to suppress NET formation and attenuate PDAC progression *in vivo*, supporting a functional link between epigenetic dysregulation and NET-mediated tumor promotion 124.

**Figure 2:**
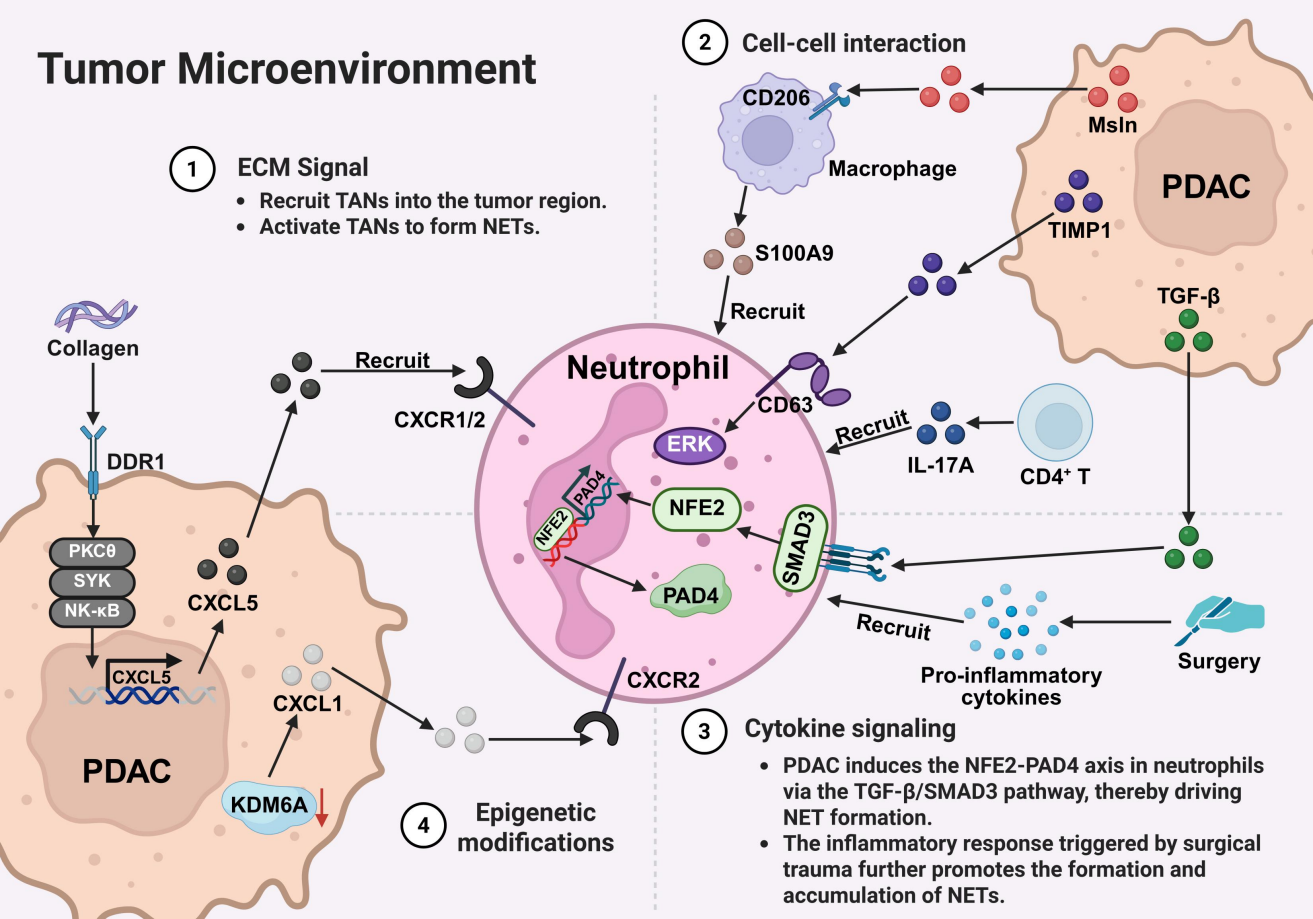
**Mechanisms regulating NET formation.** In PDAC, the TME facilitates NET formation through coordinated multi-level signaling mechanisms. These mechanisms can be categorized into four principal pathways:** (1) ECM signaling:** the collagen-rich ECM activates the DDR1 signaling pathway in tumor cells. This cascade involves the PKCθ-SYK-NF-κB axis, leading to CXCL5 upregulation. Subsequently, CXCL5 recruits TANs through CXCR1/2 and induces NET formation. **(2) Cell-cell interactions:** PDAC cell-derived Msln interacts with CD206⁺ macrophages and stimulates the release of S100A9. In parallel, tumor-derived TIMP1 and CD4⁺ T cell-derived IL-17A collectively promote neutrophil recruitment, activation, and NET formation.** (3) Cytokine signaling:** TGF-β secreted by both primary PDAC tumors and metastatic lesions, such as liver metastases, activates the NFE2-PAD4 axis in neutrophils through SMAD3 signaling, thereby promoting NET formation. Furthermore, inflammatory cytokines induced by surgical trauma contribute to additional NET generation and accumulation. **(4) Epigenetic regulation:** loss of KDM6A leads to the upregulation of chemokines, including CXCL1, which facilitates neutrophil recruitment and promotes NETosis through the CXCR2 signaling pathway. *Created with BioRender.com.

As illustrated in **Figure 2**, the formation of NETs in PDAC can be generally classified into four main regulatory pathways within the TME: ECM-driven signals, cell-cell interactions, cytokine-mediated regulation, and epigenetic alterations. These mechanisms jointly regulate the recruitment, activation, and formation of neutrophils' NETs, and exhibit distinct context-dependent characteristics. Specifically, collagen-driven activation of DDR1 is closely linked to ECM remodeling and may promote neutrophil recruitment and NET formation. At the same time, tumor-stroma interactions (such as macrophage-neutrophil crosstalk mediated by mesothelin and the TIMP1/CD63 axis) can further enhance neutrophil activation and NET release. The cytokine signaling pathways (including TGF-β signaling in the tumor microenvironment and the inflammatory response driven by IL-17) provide important regulatory levels for the phenotypic regulation of neutrophils and the accumulation of NETs. Additionally, epigenetic alterations, such as KDM6A deficiency, may indirectly promote NETosis via chemokine-mediated pathways, thereby contributing to tumor progression.

Overall, these studies suggest that NET formation in PDAC is not driven by a single pathway but rather by the combined effects of multiple intrinsic tumor factors and signals from the tumor microenvironment. However, the relative contributions of these pathways and their applicability across different disease stages still need to be clarified.

## 4. Mechanisms of PDAC progression facilitated by NETs

### 4.1 Promotion of Venous Thrombosis

Venous thromboembolism (VTE) is a common complication in patients with malignant tumors, drawing significant attention due to its high incidence and mortality rate, second only to cancer progression 125. VTE not only markedly prolongs hospitalization, reduces quality of life, and increases healthcare costs, but it is also the leading cause of death aside from tumor progression 126. Notably, the risk of VTE is closely associated with cancer type and stage, with PDAC patients exhibiting the highest incidence of VTE among all cancers, and this complication is strongly linked to poor prognosis 126-128. Emerging evidence suggests that NETs may contribute to cancer-associated thrombosis through multiple mechanisms, including the promotion of endothelial cell activation, enhancement of platelet adhesion, and activation of the coagulation cascade 129, 130. In this process, NETs may serve as a procoagulant scaffold, providing a structural foundation for thrombus formation.

Support for this mechanism comes from multiple experimental systems. *In vitro* studies show that PDAC cells, such as the human PDAC cell line (AsPC-1), can directly stimulate neutrophils to form NETs independently of ROS. This enhances platelet adhesion and promotes thrombus formation under shear stress conditions 131. *In vivo*, mouse models bearing pancreatic tumors exhibit elevated levels of circulating neutrophils, cell-free DNA (cfDNA), and CitH3. These models also show denser fibrin networks within thrombi, further supporting a role for NETs in thrombogenesis 132. Clinical observations also suggest an association between NET-related markers and thrombotic risk. For example, elevated plasma CitH3 levels have been linked to increased risk of VTE in certain cancers, including PDAC 133. In addition, PDAC patients display stage-dependent increases in phosphatidylserine (PS), PS-positive microparticles, and NETs. These changes are associated with enhanced generation of factor Xa, thrombin, and fibrin, indicating increased procoagulant activity 134.

However, the consistency and strength of these associations appear to vary across tumor types and clinical contexts. Notably, despite the high incidence of thrombosis in brain tumors, CitH3 levels do not consistently correlate with VTE risk, suggesting tumor-specific differences in NET-mediated thrombosis 133. Moreover, most mechanistic evidence is derived from *in vitro* and preclinical models, whereas clinical data are largely correlative, limiting conclusions regarding causality. In addition, the predictive value of NET-related biomarkers, such as CitH3, in PDAC remains to be validated in larger, well-controlled patient cohorts.

### 4.2 Facilitation of Tumor Metastasis

PDAC has garnered significant attention due to its high propensity for widespread metastasis and extremely high mortality rates 135, 136. The liver is the most common site of distant metastasis 137. Within the TME, CAFs, as the predominant stromal component, exhibit remarkable heterogeneity and phenotypic plasticity 138. In the PDAC TME, NETs may interact with CAFs and potentially participate in various pro-tumor processes. Studies have shown that murine PDAC cells (PCCs) can promote neutrophil recruitment and induce the formation of NETs, although the specific molecular mechanisms underlying this process remain incompletely elucidated. Conversely, deoxyribonuclease I (DNase I)-mediated degradation of NETs has been shown to impair the recruitment of activated CAFs to micrometastatic sites. These findings suggest that NETs may contribute to CAF activation and promote PDAC liver metastasis by modulating tumor-stroma interactions 139. Notably, the interaction between NETs and CAFs appears to be bidirectional. Using three murine tumor models (F10 melanoma, PDAC, and lung adenocarcinoma), Munir et al. reported that CAF-secreted beta-amyloid (Aβ) can form microaggregates within the TME, which may promote local NET formation. These aggregates may also enter the circulation and stimulate NET release from both circulating and bone marrow-resident neutrophils 140. Collectively, these findings suggest the existence of a potential NET-CAF interaction network that may contribute to a positive feedback loop in tumor progression. However, although this mechanism has been observed in multiple tumor models, including PDAC, it may represent only one of several possible crosstalk pathways between CAFs and NETs, and its relative importance in human PDAC remains to be further validated.

In addition to stromal regulation, NETs may directly modulate tumor cell behavior by promoting EMT, a key process underlying invasion, metastasis, and therapy resistance 141. Mechanistically, NETs have been shown to activate pro-inflammatory and oncogenic signaling pathways, including interleukin-1 beta (IL-1β)-EGFR/ERK signaling, thereby enhancing tumor cell migration and invasion. NETs may also activate the STING pathway in tumor cells, leading to increased IL-8 production, which in turn promotes EMT and tumor invasiveness 142. Conversely, STING-driven IL-8 release can activate neutrophils via the MEK/MAPK/ROS signaling axis, further enhancing NET formation and establishing a reinforcing feedback loop 143. Although pro-metastatic effects of NETs have been consistently observed *in vitro* and in animal models, most mechanistic evidence remains preclinical. Whether these pathways operate similarly in human PDAC and the relative contribution of CAF-dependent versus EMT-driven mechanisms across different metastatic contexts remains to be fully established. These findings suggest that NETs represent an important but context-dependent regulator of PDAC metastasis, warranting further mechanistic and translational investigation.

### 4.3 Enhancement of Chemoresistance

Drug resistance remains one of the major obstacles to the long-term survival of cancer patients 144. Even targeted therapies, which demonstrate significant clinical efficacy, often fail to achieve complete and durable tumor remission due to the emergence of resistance mechanisms, ultimately leading to disease progression or relapse 145. Chemoresistance is particularly prominent in PDAC, primarily attributable to its unique TME. The TME of PDAC is characterized by extensive desmoplasia, which compromises tumor vascular perfusion, resulting in nutrient deprivation, hypoxia, and an acidic milieu. These conditions suppress anti-tumor immune cell function and confer chemoresistance 69. Although gemcitabine (GEM) monotherapy or its combination with nab-paclitaxel (gemcitabine plus nab-paclitaxel, GNP) can improve outcomes in advanced PDAC to some extent, the persistent emergence of drug resistance severely limits their clinical efficacy 146. Studies have indicated that G protein-coupled receptor class C group 5 member A (GPRC5A) plays a critical role in PDAC chemoresistance 147. Zhu et al. reported that GPRC5A-driven NF-κB activation in PDAC cells promotes CXCL8 secretion, which in turn stimulates neutrophils and induces NET formation 148. This process is accompanied by activation of the nucleotide oligomerization domain (NOD)-like receptor protein 3 (NLRP3) inflammasome in neutrophils, suggesting a potential link between inflammatory signaling and NET formation. However, the precise mechanistic link between NLRP3 activation and chromatin decondensation during NETosis remains incompletely defined 59. Functionally, NET-derived cfDNA acts on tumor cells to enhance proliferation and migration, thereby contributing to chemoresistance. These findings support a role for the GPRC5A/NF-κB/CXCL8/NLRP3/NETs-cfDNA signaling axis in PDAC progression 148. Consistent with Zhu et al., significant NET accumulation was observed in tumor tissues from PDAC patients with poor response to GEM-based chemotherapy. Mechanistically, chemotherapy-induced IL-8 expression has been associated with activation of a ROS-mediated NF-κB/STAT3 signaling pathway. IL-8 can act on neutrophils through CXCR1/2 receptors, activating downstream signaling events that promote NET formation, including ROS generation and inflammatory signaling. NETs not only enhance tumor cell survival by activating the ERK signaling pathway and regulating Bcl-2 family proteins, but also promote tumor cell proliferation, migration, and immune evasion by releasing DNA and proteases. Importantly, the CXCR1/2 inhibitor navarixin effectively blocks NET formation and restores the sensitivity of PDAC cells to GEM, thereby suppressing tumor progression 149. However, these findings are primarily derived from preclinical studies, and further validation in clinical settings is required.

**Figure 3:**
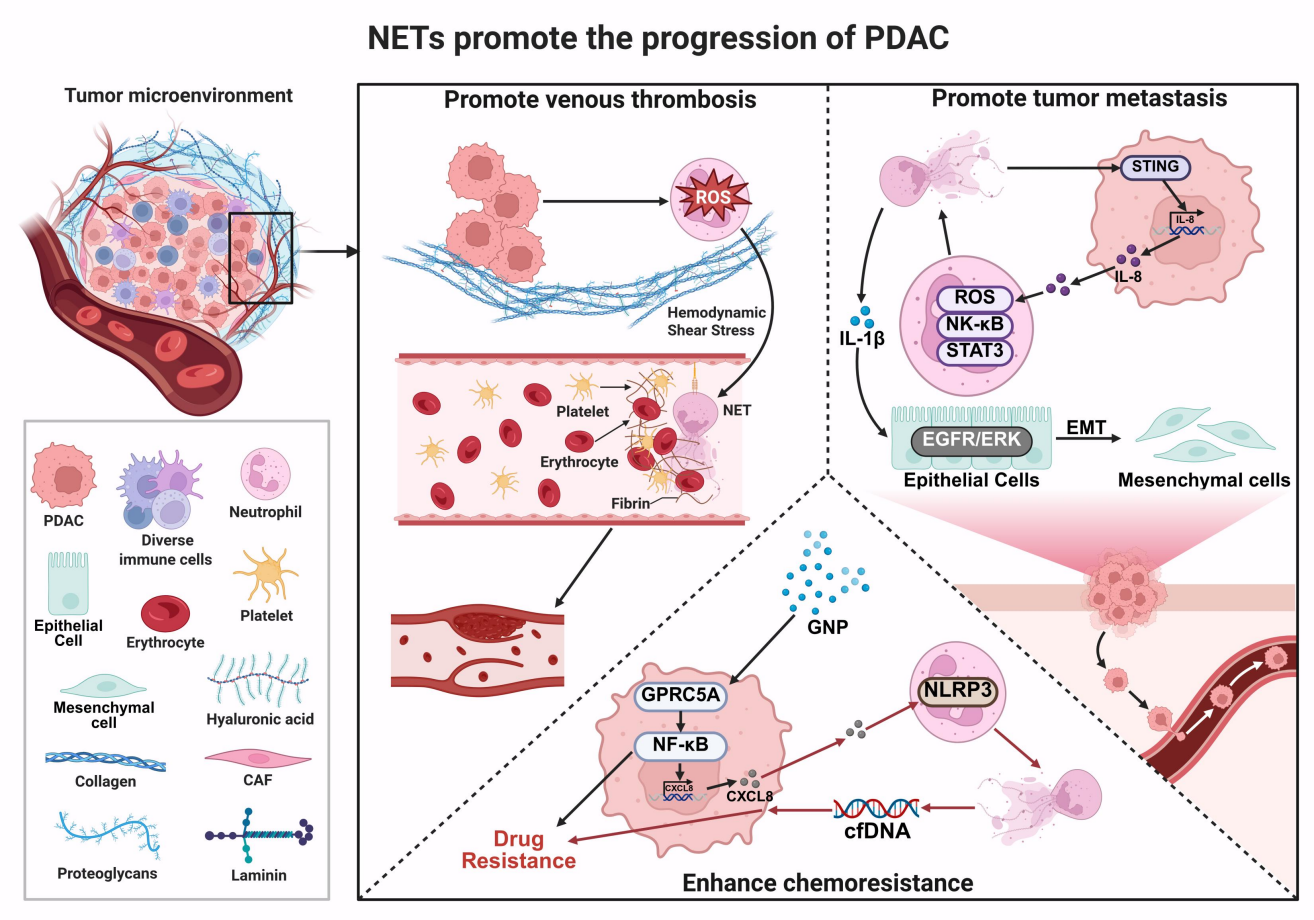
** Multilevel mechanisms by which NETs promote PDAC progression.** NETs promote tumor-associated venous thrombosis by inducing ROS production and interacting with platelets, fibrin, and erythrocytes, thereby exacerbating the hypercoagulable state and creating a favorable microenvironment for tumor cell survival and expansion. **(2)** NETs activate NF-κB, STAT3, and STING signaling pathways in tumor and immune cells, leading to the release of inflammatory mediators such as IL-8 and IL-1β. NETs also enhance PDAC invasiveness and metastatic potential by promoting EMT through the EGFR/ERK axis. **(3)** GNP and cfDNA released by NETs activate the GPRC5A-NF-κB and NLRP3 inflammasome pathways, respectively, and upregulate chemokines such as CXCL8, thereby compromising chemotherapy and targeted therapy efficacy and contributing to therapy resistance. *Created with BioRender.com.

As illustrated in **Figure 3**, NETs exert multi-layered and systemic regulatory roles in PDAC progression, spanning several key biological processes, including thrombosis, metastatic dissemination, and therapy resistance. Rather than being driven by a single pathway, NETs function as an integrative platform that tightly couples inflammatory responses with tumor progression and acts on both tumor cells and their surrounding microenvironment. Within this framework, NETs may exacerbate tumor-associated hypercoagulability by participating in coagulation-related processes. They may also enhance tumor cell invasiveness and metastatic potential through remodeling tumor-stroma interactions. In addition, NET-associated molecules and the inflammatory signals they mediate can influence tumor cell survival and stress responses, thereby contributing to reduced treatment sensitivity and the development of resistance. Collectively, these findings suggest that NETs act as a key functional hub linking inflammation, thrombosis, and malignant progression in PDAC.

Although accumulating evidence indicates that NETs play a critical role in PDAC progression, most current findings are derived from preclinical models, and their stage-specific contributions and clinical relevance remain to be fully defined. In particular, the temporal and contextual roles of NET-mediated processes-including tumor progression, metastasis, and chemoresistance-require systematic clarification. To address this, we have summarized the major NET-associated mechanisms and their functional contributions across different stages of PDAC progression **(Table 3)**.

## 5. Therapeutic Targeting of NETs in PDAC

In the progression of PDAC, NETs play a critical role. By inhibiting key molecules involved in NET formation (such as PAD4 and MPO) or blocking their downstream effects (e.g., NET-mediated procoagulant and proinflammatory signaling pathways), it is possible to delay tumor progression, reduce the risk of VTE, and enhance chemosensitivity **(Table 4)**.

### 5.1 Targeting Key Components of NETs

#### 5.1.1 Disruption of NET Structure by DNA Degradation

Numerous studies have demonstrated that degrading the DNA backbone of NETs with DNase I effectively disrupts their web-like structure, thereby exerting therapeutic effects in various disease models, including inflammatory bowel disease (IBD) 150, chemotherapy-induced peripheral neuropathy 151, gastric cancer 152, 153, HCC 154-156, breast cancer 157, and melanoma 158. Obesity is recognized as a significant epidemiological risk factor for PDAC 159-161. Studies have shown that plasma NET levels are significantly elevated in both obese individuals and obese mouse models 162, 163. Metformin, a widely used antidiabetic drug and anti-aging intervention, has recently gained increasing attention in anti-tumor research 164-166. Wang et al. found that in obese mice, the number of intrapancreatic adipocytes increased, and pancreatic steatosis was aggravated. Factors secreted by adipocytes promoted neutrophil recruitment and NET formation, which further exacerbated pancreatic steatosis and the development of murine pancreatic intraepithelial neoplasias (mPanINs). Enhanced NET generation was identified as a key mechanism by which neutrophils promote mPanIN progression in obese mice. Given that DNA constitutes the primary structural scaffold of NETs, the researchers employed a combination of metformin and DNase I in both *in vivo* and *in vitro* experiments. This intervention significantly reversed the obesity-and NET-mediated pro-tumor effects, suppressed NET-induced EMT, and inhibited TLR4 activation, thereby effectively attenuating tumor initiation and progression 78. These findings suggest that targeted degradation of NET-DNA may represent a novel therapeutic strategy for intervening in early-stage obesity-associated PDAC. However, the clinical application of DNase I may be limited by its short half-life, rapid systemic clearance, and suboptimal tissue penetration 167, 168. In addition, degradation of the NET-DNA scaffold may disrupt the structural organization of NETs and facilitate the release of associated proteins, including histones and NE which have well-established pro-inflammatory and cytotoxic effects 169, 170.

#### 5.1.2 Inhibition of PAD4-Dependent NETosis

The formation of NETs relies on the nuclear localization signal and citrullination activity of PAD4 42, 171. Numerous studies have confirmed that inhibiting PAD4 effectively blocks NET generation, thereby delaying the progression of various diseases. For instance, suppression of PAD4-mediated NET formation alleviates hypoxic-ischemic brain injury in neonatal mice 172. Additionally, downregulation of PAD4 expression through zinc supplementation also reduces NET production 173. In CRC tissues and cells, both mRNA and protein levels of PAD4 are significantly upregulated, and its high expression is closely associated with poor patient prognosis. Application of the PAD4 inhibitor GSK484 enhances the radiosensitivity of CRC cells: on one hand, it induces cell death by promoting DNA double-strand breaks; on the other hand, it delays tumor progression by inhibiting NET formation *in vivo* 174. Notably, chloroquine (CQ) was first used for the prevention and treatment of malaria in the 1950s. Subsequently, hydroxychloroquine (HCQ), due to its higher safety profile and fewer side effects, has been widely adopted as a substitute for CQ 175, 176. One of their common mechanisms of action involves the inhibition of autophagy 177. However, a study by Ivey et al. was the first to demonstrate a novel mechanism of action of CQ and HCQ in NET formation: both can directly bind to and inhibit PAD4 activity in a dose-dependent manner. Molecular simulation (iTASSER) analysis indicated that CQ forms hydrogen bonds with the Arg639 residue of PAD4, while HCQ is predicted to interact with Trp347, Ser468, and Glu580 residues. It is encouraging that, via this mechanism, both CQ and HCQ reduce circulating levels of CitH3 and significantly suppress NET formation in patient-derived PDAC samples, thereby attenuating disease progression. Importantly, CQ and HCQ are already FDA-approved and orally bioavailable, making them promising candidate drugs for clinical strategies aimed at inhibiting NET formation 178.

#### 5.1.3 Targeting MPO-Mediated NET Formation

MPO is a key component of NETs and can interact with NE to promote chromatin decondensation and subsequent NET release 54, 179. Group A streptococcus (GAS) produces various peptides and superantigens that can induce tumor regression 180, 181. Among these, the streptococcal collagen-like protein 1 (Scl1), an important adhesin molecule, specifically recognizes and binds to type III fibronectin (Fn) repeats in the tumor ECM, thereby exerting antitumor effects¹⁸⁷. It is worth noting that Scl1 is expressed in the vast majority of experimentally tested GAS strains 182. Research by Henderson et al. demonstrated that in a PDAC-bearing mouse model (KPC mice), injection of GAS significantly suppressed the growth of subcutaneous PDAC tumors, and this inhibitory effect depended on Scl1 expressed by GAS. Mechanistically, Scl1 reduces the activity of MPO, a crucial component in NET formation, effectively inhibiting neutrophil infiltration, NET generation, and PDAC progression both *in vivo* and *in vitro*. This discovery provides a new therapeutic rationale for targeting pro-tumorigenic NETs with recombinant Scl1 (rScl1.1) 183.

### 5.2 Emerging Therapeutic Approaches Targeting NET-Associated Pathways

#### 5.2.1 Autophagy Inhibition

The receptor for advanced glycation end products (RAGE) can mediate neutrophil autophagy, thereby promoting NET formation. Studies have shown that RAGE deficiency or treatment with autophagy inhibitors (such as CQ) significantly suppresses NET generation 184. In studies involving both mouse models of PDAC and clinical patient samples, Boone et al. found that treatment with the autophagy inhibitor CQ significantly reduced NET formation. This suppression reduced NET-mediated platelet aggregation and the release of circulating tissue factor (TF), ultimately alleviating PDAC-associated hypercoagulability. Subsequently, in a randomized controlled trial conducted by the same group, perioperative treatment with HCQ, an inhibitor of NET formation, was associated with a reduction in the incidence of VTE in PDAC patients, from 30% to 9.1%. Although this difference did not reach statistical significance, the observed trend supports targeting autophagy to suppress NETs and potentially reduce thrombotic risk 185.

#### 5.2.2 Targeting NET-Derived HMGB1

EMT is recognized as a key mechanism through which NETs promote tumor metastasis and invasion 41. HMGB1, a DNA-binding protein predominantly located in the nucleus, can be actively released into the extracellular space upon cellular stimulation, where it participates in DNA damage repair and maintenance of genomic stability 186. HMGB1 released from NETs induces EMT in cancer cells, thereby significantly enhancing PDAC cell migration and invasion. Interestingly, in a mouse model of PDAC, administration of thrombomodulin (TM) degrades HMGB1, which subsequently inhibits, blocks the EMT process, and ultimately impedes cancer cell metastasis. This finding indicates that targeting the argeting the NET-HMGB1 pathway may represent an effective therapeutic strategy to improve the prognosis of patients with PDAC 40.

#### 5.2.3 Inhibition of Cytokine-Induced NETs

Inflammatory responses can induce the formation of NETs, which in turn exacerbate inflammation, establishing a positive feedback loop 187. During the development and progression of PDAC, IL-17 is predominantly secreted by CD4^+^ T cells and γδ T cells and promotes the initiation and progression of precancerous lesions 114. Studies have shown that inhibiting neutrophils or blocking PAD4-dependent NET formation can neutralize the pro-tumor effects of IL-17. In human PDAC, high expression of IL-17 and PAD4 is associated with increased NETs production and correlates with poorer patient prognosis 94. Therefore, targeting the IL-17 signaling pathway may represent a potential therapeutic strategy for PDAC. Furthermore, inhibiting IL-8 and its downstream pathways, such as the CXCR1/2 receptors, has been shown to suppress NET formation, thereby delaying the progression of PDAC 149.

#### 5.2.4 Targeting ARG1-Mediated Immunosuppression

The study by Canè et al. revealed that NETs in patients with PDAC are enriched with human ARG1. This suggests that NETs serve as a critical platform for arginine metabolism-mediated immunosuppression. Within the confined microdomain of NETs, CTSS cleaves hARG1, generating a molecular form with enhanced enzymatic activity under physiological pH. The heightened hARG1 activity significantly suppressed T cell proliferation. This effect could be reversed by using hARG1-specific antibodies or inhibiting CTSS-mediated cleavage. Small-molecule inhibitors, however, proved ineffective. Further investigations demonstrated that blocking ARG1, in combination with immune checkpoint inhibition, restored CD8⁺ T cell function. This approach promoted the infiltration and activation of tumor-specific CD8⁺ T cells in PDAC-bearing mouse models, thereby enhancing the efficacy of immunotherapy 41.

Currently, numerous therapeutic strategies targeting NETs for the treatment of PDAC remain under investigation. Although CQ and HCQ have received FDA approval for other indications, their application in PDAC through NETs targeting remains exploratory, primarily confined to preclinical studies and early-phase clinical evaluations, and has not yet been established as a standard clinical treatment strategy. Beyond CQ and HCQ, the efficacy and mechanisms of action of the majority of other related inhibitors are predominantly supported by *in vitro* and murine *in vivo* studies 178, 184. However, these strategies remain at an early stage of development.

### 5.3 Challenges and Clinical Translatability

While targeting NETs has attracted considerable interest as a therapeutic strategy for PDAC, translating this approach into the clinic remains a substantial challenge. A primary concern is the essential role NETs play in host defense; systemic suppression of NET formation could heighten the risk of infection 187, 188. Additionally, many existing interventions lack precision. Strategies aimed at degrading extracellular DNA, for instance, may also affect non-NET-associated DNA or related signaling cascades, leading to off-target effects. The characteristically dense stromal matrix and elevated interstitial pressure in PDAC further aggravate pharmacokinetic challenges, including short drug half-life and poor tissue penetration. The considerable heterogeneity observed in NETs also implies that a single therapeutic modality is unlikely to be effective across diverse disease stages or patient subgroups 189. Perhaps most critically, the majority of NET-targeting strategies for PDAC remain in the preclinical phase, supported mainly by evidence from *in vitro* studies and mouse models, with their efficacy and safety yet to be established in human trials 190.

Consequently, advancing NET-targeted therapy for PDAC will depend on two key efforts: developing more selective targeting strategies and conducting rigorous clinical studies to validate their therapeutic potential.

## 6. Conclusion and Future Perspectives

In summary, mounting evidence underscores the significant regulatory role of NETs in PDAC progression. Despite recent advances in therapeutic approaches for PDAC, its overall mortality remains high, reflecting in part an incomplete understanding of the unique TME and the pathological mechanisms underlying NET formation in this disease. The TME in PDAC is characterized by dense fibrotic stroma, chronic inflammation, and profound immunosuppression, which collectively drive NET formation through multiple interconnected signaling pathways, further promoting malignant progression. Therefore, NETs should not be considered merely downstream by-products of inflammation but rather redefined as critical regulatory hubs within the PDAC tumor ecosystem.

Although several preclinical studies targeting NETs and their associated proteins have shown potential in suppressing PDAC progression and metastasis, critical questions remain unresolved: (1) The functional heterogeneity and cellular origins of NETs are still not fully defined. Available evidence indicates that NET composition is stimulus-dependent; different inducing conditions can alter protein makeup and post-translational modification profiles, thereby conferring distinct biological functions. Moreover, the PDAC TME harbors diverse stimuli that may drive distinct polymorphonuclear neutrophil subsets to form structurally and functionally heterogeneous NETs, exerting divergent effects on tumor progression. For instance, complement component C5a can induce NET formation from polymorphonuclear myeloid-derived suppressor cells (PMN-MDSCs), further highlighting significant heterogeneity in the cellular origins of NETs 191. Although this finding does not originate directly from PDAC-related research, it provides an important reference for understanding the diversity of cellular sources of NET formation in PDAC. Therefore, it is essential to systematically delineate the key NET-producing cell populations and their functional characteristics in PDAC progression. (2) The composition and structure of NETs require systematic characterization. NETs are complex, with diverse associated markers, including NET-DNA, CitH3, and MPO. Different detection markers may lead to discrepancies in NET level assessment. Thus, establishing a standardized, reproducible quantitative assay for NETs is crucial for advancing their clinical translation. (3) The upstream regulatory network driving NET formation in PDAC has not been systematically elucidated. While studies suggest roles for ECM remodeling, stromal-immune cell interactions, and cytokine signaling, further research is needed to clarify the precise molecular links between these tumor-derived signals and NET induction. (4) Translational applications remain challenging. From a translational perspective, developing reliable NET-related biomarkers and effective, safe intervention strategies is difficult. Current approaches mainly focus on inhibiting NET formation or degrading extracellular DNA, but they still face limitations in specificity and safety. Future research should emphasize the development of precise interventions targeting specific NET components or regulatory pathways, combined with biomarker-based patient stratification and rational combination therapies, to enhance the clinical translational value of NET-targeted strategies.

Overall, addressing these key questions will not only deepen our comprehensive understanding of NET regulatory networks in PDAC but also provide an important theoretical foundation for developing more precise and effective therapeutic approaches.

## Figures and Tables

**Table 1:** Comparison of granulocyte-derived extracellular traps.

ETs	First description	Major components	Formation Mode	ROS dependence	Reference
NETs	2004	Nuclear or mitochondrial DNA, histones, MPO, NE	lytic NETosis (cell death); vital NET formation (cell survives)	Largely ROS-dependent	12
EETs	2008	Nuclear or mitochondrial DNA, major basic protein (MBP), eosinophil cationic protein (ECP)	Mainly non-lytic	Primarily ROS-dependent.	192
BETs	~2014	Nuclear DNA, histones, basophil granule proteins	Not fully elucidated	Unclear/context-dependent	193

**Table 2:** Mechanistic roles of NET components.

NETs components	Key pathway/target	Key findings	Disease model
NET-DNA	CCDC25/β-parvin/RAC1/CDC42	Induces cytoskeletal remodeling and promotes directional tumor cell migration	CRC and metastasis models 14
NET-DNA	TMCO6 (CD8⁺ T cells)	Impairs TCR signaling and suppresses CD8⁺ T cell anti-tumor function	HCC 16
NE	ERK signaling pathway	Promotes tumor cell migration and liver metastasis formation	CRC liver metastasis model 34
CitH3	Remains unclear	CitH3-positive NETs are associated with poor postoperative survival and serve as an independent prognostic factor	EHCCs 38
PD-L1	PD-1 (T cells)	Induces T cell exhaustion and promotes immune evasion	TME studies 39
HMGB1	EMT-associated pathways	Promotes tumor metastasis through induction of EMT	PDAC 40
ARG1	CTSS interaction	Enhances immunosuppression and promotes PDAC progression	PDAC 41
CTSG	Protease-mediated matrix remodeling	NET-associated CTSG promotes tumor invasion and metastasis and is associated with poor prognosis	HCC 35

**Table 3:** Stage-specific roles of NETs in PDAC progression.

Disease stage	Spatial/biological context	Key NET-associated mechanisms	Functional outcomes
Early stage (tumor initiation/TME activation)	Primary TME (inflammation, hypoxia)	CXCL chemokines/IL-17 axis drive neutrophil recruitment/activation; DDR1/TIMP1 promote NET formation	Amplifies inflammation and TME remodeling, facilitating early tumor progression 94, 102, 109
Early-intermediate stage (local progression)	Tumor vasculature and systemic circulation	NETs act as procoagulant scaffolds promoting platelet adhesion and coagulation cascade activation; phosphatidylserine-dependent coagulation	Induction of hypercoagulability and VTE, contributing to vascular complications 133, 134
Metastatic stage (dissemination and colonization)	Pre-metastatic niches (e.g., liver) and CAF-rich regions	NET-CAF interactions; stromal-driven NET induction; Aβ signaling; IL-1β-EGFR/ERK and STING-IL-8 axes	Promotes EMT, tumor migration, invasion, and metastatic dissemination 142, 143
Advanced stage (therapy and resistance)	Fibrotic and immunosuppressive TME	GPRC5A/NF-κB/CXCL8/NLRP3 axis; IL-8-CXCR1/2 pathway; ERK/Bcl-2 signaling; chemotherapy-induced NETs	Enhances tumor survival, immune evasion, and chemoresistance 148, 194
Across stages (systemic and local effects)	Systemic circulation and tumor immune microenvironment	Continuous release of NET-derived cfDNA, proteases, and inflammatory mediators	Sustains immunosuppression and tumor-promoting microenvironment 132

**Table 4:** Inhibitors and therapeutic agents targeting NETs in PDAC.

Mechanism of Action	Target	Drug/Strategy	Research Type	Major Findings	Reference
Digestion of NETs	DNA backbone of NETs	DNase I	*In vivo*	Inhibition of NET formation to reduce metastasis	139
Digestion of NETs	DNA backbone of NETs	DNase I	*In vivo* and *in vitro*	DNase I inhibits NETs -mediated EMT	78
Inhibit MPO activity	MPO	rScl1.1	*In vitro*	Suppression of MPO activity inhibits the growth of pancreatic metastatic tumors	183
Inhibit PAD4 activity	PAD4	CQ; HCQ	*In vivo*; *Clinical trial*	Reducing the levels of circulating CitH3 significantly inhibits PDAC progression	178
Inhibit TLR4-dependent NETs	IL-1β	Metformin	*In vivo* and *in vitro*	Inhibits TLR4 activation and suppresses NET formation	78
Inhibition of autophagy- or PAGE-dependent NET formation	Autophagy	CQ; HCQ	*In vivo* and *in vitro*; *Clinical trial*	Suppressing NET generation and its associated hypercoagulability	184
Inhibit IL-17-induced NETs	CXCL1-CXCR2 axis	IL-17 and IL-17RA blocker	*In vivo* and *in vitro*	Synergizes with immune checkpoint inhibitors (anti-PD-1/CTLA-4) to enhance antitumor immunity	94
Inhibition of NET-derived hARG1	hARG1	hARG1-specific monoclonal antibody (mAb)	*In vivo* and *in vitro*	Blocking NETs -driven T cell suppression	41
Inhibition of NETs-derived HMGB1	HMGB1	TM	*In vivo* and *in vitro*	Targeting the NET-mediated EMT process to suppress PDAC liver metastasis	40
Blockade of CXCR2/CXCL1 axis-induced NETs	CXCL1-CXCR2 axis	Anti-CXCL1 neutralizing antibody	*In vivo*	Reduces NET formation and tumor growth in KDM6A-deficient PDAC models	124
Inhibiting IL-8-induced NETs	IL-8-CXCR1/2 axis	Navarixin	*In vivo*	Suppresses chemo-induced NETs and restores gemcitabine sensitivity in PDAC	149

## Abbreviation

Aβ: beta-amyloid
ARG1: arginase-1
AsPC-1: human pancreatic ductal adenocarcinoma cell line
BHLHE40: basic helix-loop-helix transcription factor 40
CA19-9: carbohydrate antigen 19-9
CAF: cancer-associated fibroblasts
CCDC25: coiled-coil domain containing 25
CD206: macrophage mannose receptor
CGD: chronic granulomatous disease
CitH3: citrullinated histone H3
CQ: chloroquine
CRC: colorectal cancer
CTSG: cathepsin G
CTSS: cathepsin S
C5a: complement component 5a
C5aR1: complement C5a receptor 1
CXCL1: C-X-C motif chemokine ligand 1
CXCL5: C-X-C motif chemokine ligand 5
CXCR2: C-X-C motif chemokine receptor 2
NET-DNA: DNA component of NETs
DDR: discoidin domain receptor (DDR1 and DDR2)
DNase I: deoxyribonuclease I
ECM: extracellular matrix
EMT: epithelial-mesenchymal transition
ETs: extracellular traps
EHCCs: extrahepatic cholangiocarcinomas
ECP: eosinophil cationic protein
ERK: extracellular signal-regulated kinase
FGF: fibroblast growth factor
GAS: group A streptococcus
G-CSF: granulocyte colony-stimulating factor
GM-CSF: granulocyte-macrophage colony-stimulating factor
GEM: gemcitabine
GNP: gemcitabine plus nab-paclitaxel
GPI-AP: glycosylphosphatidylinositol-anchored protein
GPRC5A: G Protein-coupled receptor class C group 5 member A
GSDMD: gasdermin D
HA: hyaluronic acid
HCQ: hydroxychloroquine
H3K27me3: trimethylation of lysine 27 on histone H3
HCC: hepatocellular carcinoma
HGF: hepatocyte growth factor
HMGB1: high mobility group box 1
IBD: inflammatory bowel disease
IL-1β: interleukin-1 beta
IL-6: interleukin-6
IL-8: interleukin-8
IL-17: interleukin-17
IL-17RA: interleukin-17 receptor A
KDM6A: lysine (K)-specific demethylase 6A
LPS: lipopolysaccharide
MAPK: mitogen-activated protein kinase
MPO: myeloperoxidase
MBP: major basic protein
mPanINs: murine pancreatic intraepithelial neoplasias
NOX: NADPH oxidase
NE: neutrophil elastase
NETs: neutrophil extracellular traps
NFE2: nuclear factor erythroid 2
NLR: neutrophil-to-lymphocyte ratio
NLRP3: nucleotide oligomerization domain (NOD)-like receptor protein 3
PAD4: peptidylarginine deiminase 4
PDAC: pancreatic ductal adenocarcinoma
PD-1: programmed cell death protein 1
PD-L1: programmed death-ligand 1
PDGF: platelet-derived growth factor
PKC: protein kinase C
PMA: phorbol 12-myristate 13-acetate
PS: phosphatidylserine
PSCs: pancreatic stellate cells
PPAR-γ: peroxisome proliferator-activated receptor-gamma
PMN-MDSCs: polymorphonuclear myeloid-derived suppressor cells
RAGE: receptor for advanced glycation end products
ROS: reactive oxygen species
Scl1: streptococcal collagen-like protein 1
scRNA-seq: single-cell RNA sequencing
SMAD3: small mother against decapentaplegic (SMAD) family member 3
ST: spatial transcriptomics
TANs: tumor-associated neutrophils
TGF-β: transforming growth factor-beta
TF: tissue factor
TIMP1: tissue inhibitor of metalloproteinases 1
TMCO6: transmembrane and coiled-coil domains 6
TLR4: Toll-like receptor 4
TNM: tumor-node-metastasis
TME: tumor microenvironment
VEGFA: vascular endothelial growth factor A
VTE: venous thromboembolism
